# Macrophage‐mediated cholesterol handling in atherosclerosis

**DOI:** 10.1111/jcmm.12689

**Published:** 2015-10-23

**Authors:** Dimitry A. Chistiakov, Yuri V. Bobryshev, Alexander N. Orekhov

**Affiliations:** ^1^ Division of Laboratory MedicineDepartment of Molecular Genetic Diagnostics and Cell BiologyInstitute of PediatricsResearch Center for Children's HealthMoscowRussia; ^2^ Faculty of Medicine and St Vincent's Centre for Applied Medical ResearchUniversity of New South WalesSydneyNSWAustralia; ^3^ School of MedicineUniversity of Western SydneyCampbelltownNSWAustralia; ^4^ Institute for Atherosclerosis ResearchSkolkovo Innovative CenterMoscowRussia; ^5^ Laboratory of AngiopathologyInstitute of General Pathology and PathophysiologyRussian Academy of SciencesMoscowRussia; ^6^ Department of BiophysicsBiological FacultyMoscow State UniversityMoscowRussia

**Keywords:** macrophages, atherosclerosis, atherogenesis, cholesterol, lipoproteins, foam cells

## Abstract

Formation of foam cells is a hallmark at the initial stages of atherosclerosis. Monocytes attracted by pro‐inflammatory stimuli attach to the inflamed vascular endothelium and penetrate to the arterial intima where they differentiate to macrophages. Intimal macrophages phagocytize oxidized low‐density lipoproteins (oxLDL). Several scavenger receptors (SR), including CD36, SR‐A1 and lectin‐like oxLDL receptor‐1 (LOX‐1), mediate oxLDL uptake. In late endosomes/lysosomes of macrophages, oxLDL are catabolysed. Lysosomal acid lipase (LAL) hydrolyses cholesterol esters that are enriched in LDL to free cholesterol and free fatty acids. In the endoplasmic reticulum (ER), acyl coenzyme A: cholesterol acyltransferase‐1 (ACAT1) in turn catalyses esterification of cholesterol to store cholesterol esters as lipid droplets in the ER of macrophages. Neutral cholesteryl ester hydrolases nCEH and NCEH1 are involved in a secondary hydrolysis of cholesterol esters to liberate free cholesterol that could be then out‐flowed from macrophages by cholesterol ATP‐binding cassette (ABC) transporters ABCA1 and ABCG1 and SR‐BI. In atherosclerosis, disruption of lipid homoeostasis in macrophages leads to cholesterol accumulation and formation of foam cells.

## Introduction

Macrophages are key players in all stages of atherosclerosis. Initially, monocytes attracted by pro‐inflammatory signals coming from the inflamed endothelium attach to the problematic arterial sites and infiltrate the intima 1. To be attractive for monocytes and other immune cells, the problematic vascular sites should be injured or atheroprone because of the abnormal haemodynamic forces and/or accumulation of oxidized lipids in the arterial wall 1. In the subendothelial layer, monocytes differentiate to macrophages that in turn polarize to pro‐inflammatory/anti‐inflammatory phenotype depending on the local stimuli and transform to foam cells 2.

**Figure 1:**
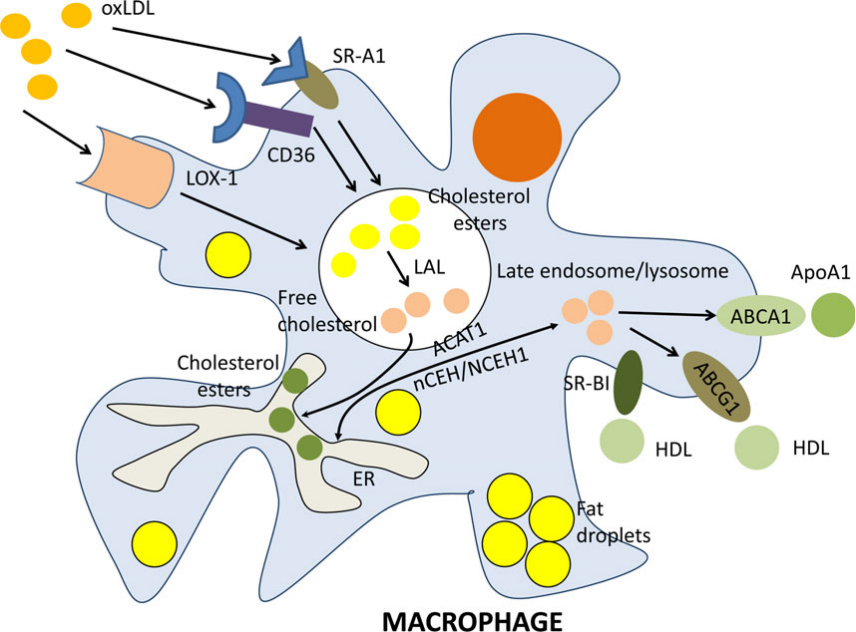
Cholesterol metabolism in macrophages. Macrophages engulf oxidized cholesterol (oxLDL) with help of several scavenger receptors (SR) including CD36, SR‐A1, and lectin‐like oxLDL receptor‐1 (LOX‐1). In late endosomes/lysosomes of macrophages, oxLDL are catabolysed. Lysosomal acid lipase (LAL) hydrolyses cholesterol esters that are enriched in LDL to free cholesterol and free fatty acids. In endoplasmic reticulum (ER), acyl coenzyme A: cholesterol acyltransferase‐1 (ACAT1) in turn catalyses esterification of cholesterol to store cholesterol esters as lipid droplets in the ER of macrophages. Neutral cholesteryl ester hydrolases nCEH and NCEH1 are involved in a secondary hydrolysis of cholesterol esters to liberate free cholesterol that could be then out‐flowed from macrophages by cholesterol ATP‐binding cassette (ABC) transporters ABCA1 and ABCG1 and scavenger receptor SR‐BI. The main acceptors of free cholesterol from ABCG1 and SR‐BI are high density lipoprotein (HDL) and from ABCA1 is apolipoprotein A1 (ApoA1). In atherosclerosis, disruption of lipid homoeostasis in macrophages leads to cholesterol accumulation and formation of foam cells.

Formation of foam cells that occurs in the initial stages of atherogenesis is a hallmark of atherosclerotic disease 3, 4. Increased uptake of oxidized low‐density lipoprotein (oxLDL) and/or reduced cholesterol efflux leads to the deposition of esterified cholesterol in the cytoplasm of macrophages and generation of foam cells 5. In macrophages, oxLDL is taken up with help of scavenger receptors (SR) such as CD36 (also known as fatty acid translocase), SR‐A1 and lectin‐like oxLDL receptor‐1 (LOX‐1) (Fig. 1) 6. Acyl coenzyme A: cholesterol acyltransferase‐1 (ACAT1) and neutral cholesteryl ester hydrolase (nCEH) are involved in the formation of cholesterol esters 7. ATP‐binding cassette (ABC) transporters ABCA1 and ABCG1 and SR‐BI contribute to the reverse transport of cholesterol from macrophages 8.

The purpose of this review is to characterize key mechanisms of cholesterol efflux, specifically in macrophages. In this work we briefly consider all constituents of the system responsible for cholesterol metabolism in macrophages.

## Cholesterol uptake

Cholesterol enters the macrophage cytoplasm through mechanisms of SR‐dependent phagocytosis and pinocytosis 9. CD36 and SR‐A are principal contributors to cholesterol uptake accounting up to 90% of oxLDL loading in macrophages 10.

### Scavenger receptor CD36

CD36 is a 88 kD glycoprotein that is a member of SR class B family 11. The human CD36 gene is located on chromosome 7q11.2 11. This receptor includes two extracellular and two transmembrane domains that flank the extracellular domain 12. CD36 binds oxLDL with high affinity. A major oxLDL‐binding site is located between amino acids (a.a.) 127 and 279, with an additional site located between a.a. 28–93 13. Interaction of oxLDL with CD36 causes endocytosis of CD36‐oxLDL complex *via* lipid raft‐dependent mechanism 13.

The pro‐atherogenic role of oxLDL depends on the abundance of CD36. Handberg *et al*. 14 found a correlation between the plasma levels of soluble CD36 (sCD36) and indices of insulin resistance, carotid atherosclerosis and fatty liver. Furthermore, increased levels sCD36 were associated with insulin resistance and higher risk of type 2 diabetes 15, 16. Elevated sCD36 levels were detected in the monocytes of patients with coronary artery disease 17 and acute coronary syndrome 18. Statin therapy reduces sCD36 concentrations and suppresses oxLDL uptake by monocytes and macrophages 19. Similarly, low molecular CD36 inhibitors were shown to reduce lipid deposits in the arterial wall and improve insulin sensitivity and glucose tolerance 20. However, patients deficient in CD36, had advanced atherosclerosis 21. Indeed, the role of CD36 in atherosclerosis is complicated and should be further studied in detail.

In macrophages, expression of CD36 is regulated by many factors. An antioxidant curcumin up‐regulates the CD36 expression through stimulating nuclear erythroid‐related factor 2 (Nrf2) 22. Astaxanthin, an oxidized carotenoid, increases CD36 expression by the activation of the peroxisome proliferator‐activated receptor‐γ (PPAR‐γ) 23. Palmitic acid stimulates monocyte production of CD36 by the induction of *de novo* ceramide synthesis 24 as ceramides reduce CD36 expression and decrease oxLDL uptake by monocytes 25. Similarly, lipopolysaccharide from the bacterium *Porphyromonas gingivalis*, a causative agent of gingivitis, up‐regulates CD36 in macrophages by stimulating c‐Jun/activator protein‐1 (AP‐1) pathway 26. Interestingly, a 12‐week dietary intake of champignon, *Agaricus blazei*, by Apolipoprotein E (ApoE)‐deficient mice resulted in elevated levels of CD36 and higher lesion instability suggesting for pro‐atherogenic properties of this mushroom 27.

Some compounds were shown to down‐regulate CD36 expression. For example, tanshinone IIA, a diterpene from a sage herb *Salvia miltiorrhiza* Bunge, reduces CD36 expression by antagonizing PPAR‐γ 28. Squalene (a component of olive oil) and endomorphin‐1 (an opioid peptide) decrease CD36 and disrupt lipid overload in macrophages 29, 30. Quercitrin, a plant pigment glycoside, also suppresses CD36 expression in macrophages by altering protein kinase C (PKC)/PPAR‐γ signalling pathway 31. Exposure of the macrophages to another flavonoid and antioxidant (kaempferol) prevents nuclear translocation of transcription factor AP‐1, thereby inhibiting expression of CD36 32. Finally, walnut that is rich in γ‐3 polyunsaturated fatty acids and antioxidants was shown to display an anti‐atherosclerotic activity in ApoE‐deficient mice by decreasing the CD36 expression 33. Indeed, CD36 expression could be nutritionally modulated such that it could represent a valuable tool for the prevention of atherosclerosis.

### Scavenger receptor A1

Scavenger receptor‐A1 (also known as macrophage SR MSR1 or CD204) belongs to the class A SR family 34. The human SCARA1 gene encoding this receptor is situated on chromosome 8p22 35. Three alternate transcripts are produced from this gene. Isoforms 1 and 2 are functional and able to mediate endocytosis of modified LDL. The isoform 3 cannot internalize oxLDL because of altered processing that leads to the localization of this isoform in the endoplasmic reticulum (ER) making it unable to perform endocytosis. This isoform suppresses the activity of the isoforms 1 and 2 when co‐expressed, thereby acting as a negative regulator of SR‐A1 expression in macrophages 36. SR‐A1 functions as a homotrimer consisting of three 77‐kD glycosylated subunits.

In macrophages, SR‐A1 is involved in the uptake of modified LDL. In ApoE mice, SR‐A1 knock‐down reduces the generation of foam cells and atherosclerosis progression 37. In LDL receptor‐deficient mice, inhibition of either CD36 or SR‐A1 alone had atheroprotective effect. However, suppression of both SRs showed no positive effect on atherosclerosis suggesting that compensatory activation of these receptors is sufficient for the intake of modified LDL 38. Similarly, in hyperlipidemic ApoE‐deficient mice, deletion of either CD36 or SR‐A1 significantly reduces lipid accumulation in macrophages, but does not diminish atherosclerosis 39. These data suggest for the existence of alternative mechanisms of lipid uptake by macrophages that are independent of CD36 or SR‐A1.

Like CD36, expression of SR‐A1 could be influenced by a variety of modulators. Pro‐inflammatory cytokines such as tumour necrosis factor α (TNF‐α) and interleukin‐6 (IL‐6) promote SR‐A1 expression by activation of transcription nuclear factor (NF)‐κB 40. Indeed, pharmacological inhibition of both cytokines decreases oxLDL accumulation and foam cell formation 41. Berberine, a plant alkaloid from *Berberis* sp., was shown to have pro‐atherogenic effects on culture mouse monocytes by up‐regulating SR‐A1 and increasing the cholesterol uptake by suppressing negative cell cycle regulator phosphatase and tensin homologue (PTEN) and thereby preserving protein kinase Akt from PTEN‐dependent dephosphorylation 42.

Yang *et al*. 43 showed that voltage‐gated potassium channel Kv1.3 is involved in the modulation of activity of SR‐A1. Kv1.3 activation led to increased activity of SR‐A1 and enhanced uptake of oxLDL. Indeed, antibody‐dependent Kv1.3 blockade resulted in reduced oxLDL entrance, decreased cholesterol esterification and enhanced apoA‐I‐mediated cholesterol efflux 44. This finding may have important pharmaceutical consequences as it allows specific targeting and modulation of lipid accumulation in macrophages.

Modulators such as curcumin, polyphenolic extracts of mulberry leaves and hydrogen sulphide (H_2_S) were shown to decrease SR‐A1 levels 45. In ApoE‐deficient mice, curcumin induced SR‐A1 ubiquitination and calpain‐mediated proteolysis in macrophages 45. Mulberry‐derived polyphenols down‐regulate SR‐A1 through the suppression of PPAR‐γ 46. In the vascular system, H_2_S is mainly produced by cystathionine γ‐lyase (CSE). However, in apoE‐deficient mice, this pathway is impaired resulting in reduced H_2_S production. Deregulation of CSE‐dependent generation of H_2_S contributes to oxLDL‐mediated inflammation in macrophages 47. H_2_S possesses anti‐atherosclerotic properties by decreasing plaque size and production of intercellular adhesion molecule‐1 48. H_2_S also inhibits foam cell formation by suppressing SR‐A1 *via* K_ATP_/Erk 1/2 mechanism 49.

### Lectin‐like oxidized low‐density lipoprotein receptor‐1

Lectin‐like oxidized low‐density lipoprotein receptor‐1 (LOX‐1) is a membrane glycoprotein that contains a short N‐terminal cytoplasmic domain, a transmembrane domain, a neck region that controls receptor oligomerization and an extracellular C‐type lectin‐like extracellular domain, involved in oxLDL binding 50, 51. Human LOX‐1 is encoded by the OLR1 gene located on chromosome 12p13.2‐p12.3 52. The gene encodes several LOX‐1 isoforms of which the longest isoform contains a 273‐a.a. polypeptide 53. Another variant lacks an exon that results in a frameshift and early stop codon. Indeed, the isoform 2 is shorter than isoform 1 and contains a distinct C‐terminus 54.

Compared with CD36, LOX‐1 is able to bind moderately modified, but not fully oxidized LDL suggesting for crucial role of this receptor in initial stages of atherosclerosis 55. LOX‐1 is a major oxLDL‐binding receptor in endothelial cells, but it could be up‐regulated in macrophages in atherosclerosis 56. LOX‐1 could not be found in monocytes, but may be induced in differentiated macrophages 54.

Multiple regulators are involved in the up‐regulation of this receptor especially in inflammation. Pro‐inflammatory cytokines such as IL‐1, interferon (IFN)‐γ and TNF‐α stimulate LOX‐1 expression in macrophages 57. Modified lipids such as oxLDL and products of its hydrolysis (lysophosphatidylcholine) were shown to activate LOX‐1 expression in macrophages 51. Hypertension‐related stimuli including angiotensin II and endothelin‐1 were found to induce the expression of LOX‐1 in macrophages 58. In diabetic patients, hyperglycaemia and advanced glycation end‐products (AGEs) play a key role in the up‐regulation of LOX‐1 expression in macrophages 59, 60.

Macrophage‐specific deletion of LOX‐1 did not reveal significant changes in oxLDL uptake compared with wild‐type cells 61. Therefore, in normal conditions, impact of LOX‐1 to intake and catabolism of oxLDL by macrophages is small probably because of the high contribution of other SRs. LOX‐1 knock‐down does not greatly influence oxLDL uptake by non‐stimulated macrophages as this receptor contributes for only 5–10% of a total oxLDL intake by macrophages, whereas in pro‐inflammatory‐stimulated macrophages, the impact of LOX‐1 could increase up to 40% 61.

Monocytes were shown to differentiate to dendritic cells (DCs) 62, a common event in atherosclerosis 63. High levels of LOX‐1 expression were found in mature DCs suggesting for a significant contribution to oxLDL uptake because an antibody against LOX‐1 decreases oxLDL influx by 48% 64.

A markedly increased expression of LOX‐1 was detected in atherosclerotic lesions compared to the normal vascular tissue 55. In LDL‐deficient mice with genetic deletion of LOX‐1, a less pronounced development of atherosclerotic plaques was observed in comparison with LDL‐deficient mice fed on high‐cholesterol diet. In mice, LOX‐1 knockout was accompanied by the decreased expression of the pro‐inflammatory transcription factor NF‐κB and variable pro‐inflammatory markers 65. In contrast, ApoE‐deficient mice transgenic for LOX‐1 showed advanced atherosclerosis 66. In rabbits with experimental hypercholesterolaemia, LOX‐1 was found to be predominantly expressed in unstable lesions 67. Intraplaque LOX1 levels correlated with the expression of tissue factor and apoptosis suggesting a possible involvement of this SR to plaque instability 67. All these observations suggest the pro‐atherogenic role of LOX‐1.

## Formation of cholesterol esters

**Figure 2:**
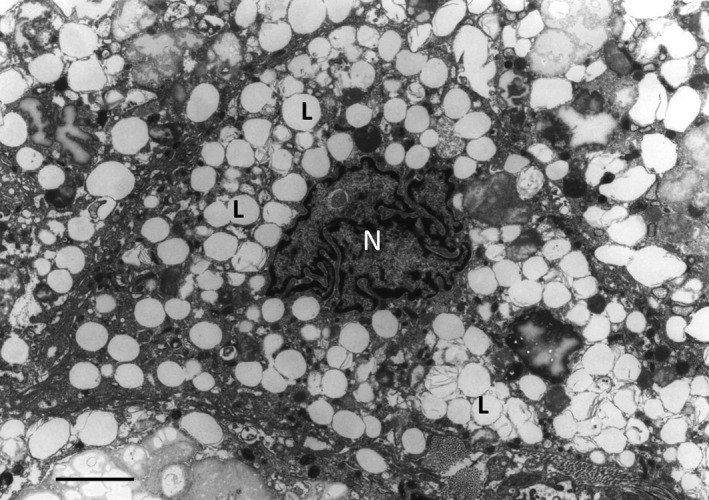
Electron micrograph showing a macrophage foam cell, the cytoplasm of which filled with a large number of ‘lipid droplets’ (L). N – nucleus. Atherosclerotic plaque tissue specimen of the human aorta. Transmission electron microscopy (TEM); scale bar = 5 μm.

The rate of cholesterol esterification could determine the fate of macrophages to be transformed to foam cells or not. After engulfment, lipoproteins are transferred to late endosomes/lysosomes where LAL hydrolyses cholesterol esters with the generation of free cholesterol. Free cholesterol in turn is *de novo* esterified by ACAT1 and stored as lipids in the ER. In case of overload with cholesterol esters, macrophages could be transformed to foam cells (Fig. 2). Indeed, cholesterol excess should be removed from the cell. The nCEH is responsible for the secondary hydrolysis of cholesterol esters 68. Free cholesterol is then transferred out with cholesterol transporters residing on the plasma membrane. Therefore, balance between hydrolysis and esterification of cholesterol plays a key role in maintaining cholesterol homoeostasis and prevention of generation of foam cells. Two enzymes, nCEH and ACAT1, are crucially involved in this mechanism.

### Acyl coenzyme A: cholesterol acyltransferase‐1

**Figure 3:**
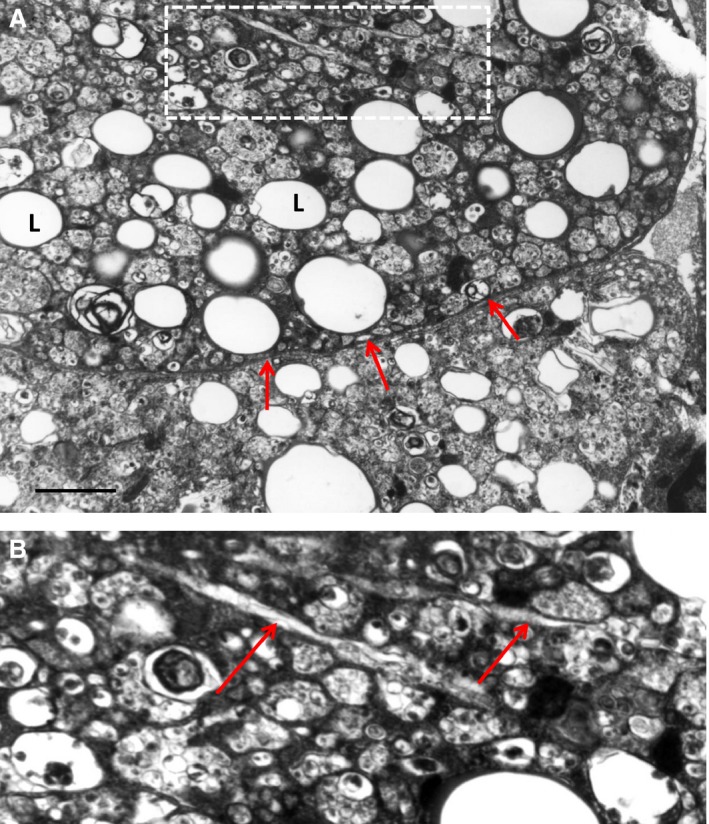
Formation of cholesterol crystals in the cytoplasm of a foam cell (**A** and **B**). (**B**) It is a detail of (**A**). L ‐ ‘lipid droplet’. In (**A**), arrows show the plasma membrane of the foam cells. In (**B**), arrows show cholesterol crystals. Atherosclerotic plaque tissue specimen of the human aorta. TEM; scale bar = 1 μm (**A**).

**Figure 4:**
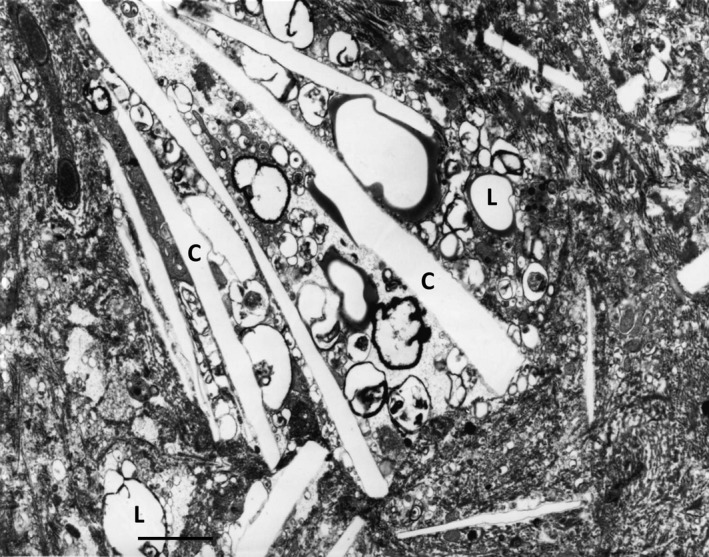
Cholesterol crystals (C) and ‘lipid droplets’ (L) located in the extracellular matrix of an atherosclerotic plaque of the human aorta. TEM; scale bar = 1 μm.

In macrophages, ACAT1 [or sterol O‐acyltransferase 1 (SOAT1)] (EC 2.3.1.26) is mainly located in the tubular rough ER 69. The human SOAT1 gene resides on chromosome 1q25 and encodes a 550 a.a. polypeptide. The enzyme contains two transmembrane domains 70. In murine peritoneal macrophages, lack of ACAT1 was reported to elevate free cholesterol levels and increase cholesterol efflux 71. In opposite, ACAT1 overproduction resulted in an enhanced accumulation of cholesterol esters followed by the formation of foam cells 72. However, other investigations showed that the role of ACAT1 in atherogenesis is not ordinary. For example, in ApoE‐deficient mice, ACAT1 suppression by a small molecular inhibitor F‐1394 had an atheroprotective effect 73. In contrast, macrophage‐specific depletion of ACAT1 in mice caused advanced atherosclerosis 74, 75. The discrepancy in these results could be explained by the non‐specific action of F‐1394 that inhibits both ACAT isoforms (ACAT1 and ACAT2) and therefore has a systemic effect 74. Human ACAT2 is mainly expressed by intestinal cells and hepatocytes where it is responsible for lipoprotein assembly 76. In addition, free cholesterol produced by ACAT1‐catalysed reaction could be seriously cytotoxic for macrophages and other vascular cells. In macrophages, free cholesterol overload could lead to the formation of cholesterol crystals (Fig. 3) that are highly cytotoxic and may not only impair intracellular cholesterol metabolism but also enhance release of pro‐inflammatory cytokines IL‐1β and IL‐18 77. These processes might progress leading to cell death, accompanied by the release and accumulation of ‘free’ cholesterol crystals in the extracellular matrix of atherosclerotic lesions (Fig. 4).

The ACAT1 expression and activity are extensively modulated by different signalling messengers. Leptin, a hormone produced by adipose tissue, stimulates ACAT1 expression through Janus‐activated kinase 2 (Jak2)/phosphatidylinositide 3‐kinase (PI3K) mechanism 78. Insulin also up‐regulates ACAT1 expression in macrophages *via* extracellular signal‐regulated kinase (Erk)/p38MAP kinase/Jnk‐dependent activation of CCAAT/enhancer binding protein α, a transcriptional regulator 79, 80. Except regulation of SR‐A1 activity, voltage‐gated potassium channel Kv1.3 is involved in the up‐regulation of ACAT1 that leads to enhanced uptake of oxLDL and accumulation of cholesterol ethers in macrophages 44.

In contrast, glucagon‐like peptide‐1 (GLP‐1) and glucose‐dependent insulinotropic polypeptide (GIP) reduce ACAT1 levels through PKA‐mediated pathway 81. Dipeptidylpeptidase 4 (DPP4) is known to degrade GLP‐1 82. Vildagliptin and other DPP4 inhibitors were shown to be atheroprotective in non‐diabetic and diabetic ApoE‐deficient mice through the activation of both incretin hormones (GLP‐1 and GIP) and restoring insulin production 83. Similarly with the suppression of SR‐A1, H_2_S utilizes the same mechanism (*e.g*. KATP/Erk 1/2‐dependent signalling) to inhibit ACAT1 expression in macrophages 49. Ghrelin, a ‘hunger’ hormone, produced by ghrelin cells in the gastrointestinal tract 84 also reduces the expression of ACAT1 by binding to the growth hormone secretagogue receptor and suppression of PPARγ 85.

### Neutral cholesteryl ester hydrolase

nCEH also known as hormone‐sensitive lipase (EC 3.1.1.79) is responsible for the hydrolysis of cholesterol esters to generate free cholesterol for further release from the cell. The human enzyme is encoded by the LIPE gene located on chromosome 19q13.2 86. Two transcripts (long and short nCEH isoforms) are produced from this gene. The long isoform is expressed in testis and other steroidogenic tissues where it converts cholesteryl esters to free cholesterol for steroid hormone production. The short isoform is present in adipocytes, macrophages and other cells 87. nCEH is a 1076‐a.a. membrane polypeptide that contains three domains (lipid‐binding, catalytic and N‐terminal). The N‐terminal domain serves as an anchor to recruit nCEH to the ER membrane where the enzyme exposes its catalytic domain to the lumen. nCEH is a glycoprotein, with three N‐glycosylated sites at N270, N367 and N389. All these sites are glycosylated, and glycosylation at N270 is essential for catalytic activity 88.

Inhibition of nCEH greatly accelerates formation of foam cells 89. Overexpression of nCEH results in the increased hydrolysis of cholesterol esters in lipid‐laden macrophages 90. However, nCEH overexpression alone without decreasing the expression of ACAT1 and stimulation of cholesterol efflux was not enough to protect against atherosclerosis 90. Transgenic mice overexpressing nCEH and cholesterol acceptor such as ApoA4 do have reduced atherosclerosis 91. Macrophage‐specific overexpression of nCEH also diminished atherosclerosis and decreased plaque necrotic core size in LDL receptor‐deficient mice 92 suggesting a critical role of macrophages in lipid handling in atherosclerosis.

Recently, a new neutral cholesterol ester hydrolase (NCEH1; also known as arylacetamide deacetylase‐like 1, AADACL1) was found 93. Human enzyme is encoded by the NCEH1 gene located on chromosome 3 94. Like nCEH, NCEH1 resides in the ER membrane and contributes to the hydrolysis of cholesterol esters in macrophages. Knockout of NCEH1 in ApoE‐deficient mice promoted the development of atherosclerosis without altering the serum lipid profile 95 suggesting the atheroprotective role of this enzyme. Indeed, both nCEH and NCEH1 are involved in the generation of free cholesterol from cholesterol esters, thereby preventing formation of foam cells from macrophages.

Insulin was found to be involved in the regulation of nCEH expression in macrophages. In the initial stages of type 2 diabetes associated with the hyperfunction of insulin‐producing β‐cells, increased levels of insulin could down‐regulate the nCEH expression in macrophages, thereby contributing to atherogenesis 96. Interleukin 33, a member of the IL‐1 cytokine family, suppresses nCEH in macrophages through the stimulation of ST‐2/NF‐κB signalling. Furthermore in ApoE‐deficient mice, IL‐33 significantly reduced accumulation of macrophages in atherosclerotic plaques and generation of foam cells 97.

## Cholesterol efflux

Free cholesterol could be removed from macrophages through active transfer mediated by cholesterol transporters or by passive transmembrane diffusion. High density lipoprotein (HDL) or ApoA1 then capture the released cholesterol. Cholesterol transporters such as ABCA1, ABCG1 and SR‐Bi play the major role in active free cholesterol efflux.

### ATP‐binding cassette transporter, ABCA1

The ABCA1, also known as cholesterol efflux regulatory protein, regulates cholesterol efflux and phospholipid homoeostasis 98. The human ABCA1 gene is located on chromosome 9q31.1 99. The 200‐kDa transporter is ubiquitously expressed throughout the body. Interestingly, mice deficient for ABCA1 and SR‐BI had severe hypocholesterolaemia mainly as a result of HDL loss, but showed no atherosclerosis because of the absence of pro‐atherogenic lipids 100. ApoA1, the major protein component of HDL, serves as an acceptor of cholesterol released by ABCA1 101. Unexpectedly, in LDL receptor‐deficient mice, hepatic ABCA1 overexpression led to the deposition of pro‐atherogenic lipids and advanced atherosclerosis because of the enhanced transfer of HDL cholesterol to LDL and delayed catabolism of cholesterol‐enriched LDL 102.

As a result of the key role in cholesterol reverse transport, ABCA1 expression is controlled by various regulators and bioactive molecules. In macrophages, the expression of ABCA1 is regulated by liver X receptor α (LXRα), a nuclear transcription factor 103. Quercetin was observed to stimulate LXRα through the activation of PPARγ that finally results in the up‐regulation of ABCA1 transcription and increased cholesterol efflux from macrophages 104. Apelin‐13, a vasoactive peptide, enhances cholesterol out‐flow from foam cells through the stimulation of PKCα and inhibition of calpain, a protease that is involved in ubiquitination‐mediated ABCA1 degradation 105, 106. Similarly, various proteasome inhibitors and ApoA1, an acceptor of cholesterol transferred by ABCA1, restore cholesterol efflux in macrophages by suppression of ABCA1 degradation 107, 108. S‐allylcysteine, a major garlic extract constituent, possesses the atheroprotective activity by increasing ABCA1 expression through unknown mechanism 109. However, lipid‐lowering properties of S‐allylcysteine appear to play a secondary role in a summary of its anti‐atherogenic effects as this cysteine derivative only moderately inhibited lipid accumulation in macrophages 110. The main beneficial effects of S‐allylcysteine on macrophage function in atherosclerosis are inhibiting inducible nitric oxide synthase and suppressing the production of hydroxyl radical, thereby suggesting for the primary role of the antioxidant activity 111.

Unsaturated free fatty acids such as palmitic acid or linoleic acid down‐regulate ABCA1 expression in macrophages by epigenetic repression of LXR genes and in LXR‐independent post‐translational level involving ABCA1 phosphorylation and destabilization by protein kinase Cδ that induces degradation of the transporter 112, 113. Pro‐inflammatory cytokines IL‐12 and IL‐18 suppress ABCA1 expression through IL‐18 receptor/NF‐κB‐mediated induction of zinc finger protein 202 (ZNF202), a transcriptional repressor 114. MicroRNA (miR)‐26 that is critically involved in the regulation of vascular smooth muscle cell differentiation 115 was shown to target LXRα and therefore down‐regulate ABCA1 expression 116. MiR‐144 directly decreases ABCA1 mRNA levels in macrophages and liver as two miR‐144‐binding sites were found in the 3′ untranslated region of the ABCA1 mRNA 117. Indeed, development of miR‐26 and miR‐144 inhibitors has a therapeutic potential to suppress the formation of foam cells in atherosclerosis.

### ATP‐binding cassette transporter ABCG1

This transporter transfers cholesterol to HDL particles, but not to ApoA1. The human ABCG1 gene is mapped to chromosome 21q22.3 118. The gene contains alternative start codons that result in the production of multiple transcripts in a tissue‐specific manner 119. In LDL receptor‐deficient mice, ABCG1 knock‐down inhibition, results in a moderate rise of atherosclerotic plaques 120. In contrast, a study shows the atheroprotective effect of genetic deletion of ABCG1 in the LDL receptor‐deficient mouse 121. Indeed, as ABCA1 is a primary cholesterol transporter in macrophages and it could compensate lipid efflux.

In fact, the regulation of expression of ABCA1 and ABCG1 is shared between LXRα and LXRβ that control tissue lipid intake 8. Notably, ABCG1 expression could be modulated by dietary components. Cultured macrophages exposed to extra‐virgin olive oil developed increased ABCG1 expression and enhanced lipid overflow 122. Cineole, a monoterpenoid of *Eucalyptus* sp. was shown to stimulate ABCG1 expression *via* activation of LXR receptors 123. Cyanidin‐3‐O‐β‐glucoside (Cy‐3‐G), an anthocyanin derived from blackberry, blueberry, bilberry, cranberry and other herbs is known to have remarkable atheroprotective properties 124. In the intestine, Cy‐3‐G could be converted by gut microflora to protocatechuic acid (PCA), a bioactive metabolite that is able to reduce expression of miR‐10b, which represses ABCA1 and ABCG1 125. Except for inhibiting lipid accumulation in macrophages, PCA exhibits anti‐inflammatory properties by decreasing subendothelial monocyte infiltration and recruitment in ApoE‐deficient mice 126. Therefore, regular intake of a proper food could prevent lipid misbalance and cholesterol deposition in macrophages and other cells.

### Scavenger receptor BI

Scavenger receptor‐BI is responsible for cholesterol transfer to HDL. The human receptor is encoded by the SCARB1 gene located on chromosome 12g24.31 44. Two SR‐BI isoforms could be translated from the human SCARB1 gene. The longest isoform contains 509 a.a., with a C‐terminal distinct from the shortest isoform (506 a.a.) 127. Both isoforms have two transmembrane domains, two short cytoplasmic tails and a large extracellular loop 128.

In ApoE‐deficient mice, macrophage‐specific deletion of SR‐BI resulted in advanced atherosclerosis, with an 86% increase in average lesion area. Overexpression of SR‐BI delayed formation of atherosclerotic plaques in ApoE‐deficient mice 129. However, in LDL receptor‐deficient mice, macrophage‐specific overproduction of SR‐BI protected against advanced atherosclerosis, but contributed to early plaque formation 130. The extraordinary role of SR‐BI in atherosclerosis could be explained by ability of this transporter to move cholesterol bidirectionally 131. At early atherosclerosis stages, SR‐BI in macrophages acts like SR‐A1 to conduct cholesterol and phospholipid influx to decrease excessive serum cholesterol levels. Furthermore, SR‐B1 was shown to block ABCA1‐dependent cholesterol efflux in macrophages suggesting for distinct and competing roles of these transporters in mediating cholesterol flux 132. Local HDL phospholipid composition could greatly influence the reverse cholesterol transport, with increased phosphatidylcholine enrichment of HDL that stimulates cholesterol efflux 131.

Numerous bioregulators modulate SR‐BI expression. Dietary components such as polyphenol resveratrol and 13‐hydroxy linoleic acid activate PPARγ that in turn up‐regulates LXRα and SR‐BI 133, 134. Similarly, caffeic acid and ferulic acid, two major phenolic acids of coffee, increase cholesterol efflux from macrophages by stimulation of SR‐BI and ABCG1, *e.g*. carriers that deliver cholesterol to HDL, but not to ApoA1 135. In contrast, pappalysin‐1 (or pregnancy‐associated plasma protein A; PAPPA), a metalloproteinase that cleaves insulin‐like growth factor binding proteins could decrease expression of ABCA1, ABCG1, and SR‐BI by inhibiting IGF‐I‐mediated (IGF/PI3‐K/Akt) stimulation of LXRα 136.

## Concluding remarks

Macrophages are master regulators of plasma lipid balance being involved in ingestion and storage of excessive lipids that could be then released in a case hypolipidaemia. However, in atherosclerosis, there is a deregulation of cholesterol uptake and reverse transport by macrophages. OxLDL, which is a major source of cholesterol, contributes to the suppression of cholesterol efflux, whereas expression of SRs especially LOX‐1 becomes significantly up‐regulated. Indeed, it is not surprising that the genetic deletion of either SR‐A1 or CD36 has no beneficial effects in ApoE‐deficient mice 38, 39 because oxLDL uptake by macrophages could be compensated by SR‐BI (in early atherosclerosis) and LOX‐1.

It should be stressed that lipid metabolism in macrophages could be modulated by multiple dietary factors that provide a good option to prevent lipid accumulation or improve macrophage function in atherosclerotic patients. For example, several polyphenolic acids derived from blueberries were shown to efficiently suppress generation of foam cells through the down‐regulation of CD36 and stimulation of ABCA1 137.

Targeting components of cholesterol uptake/esterification/efflux should be of great therapeutic value. Statins (lipid‐lowering agents) were shown to possess a wide range of anti‐inflammatory activities including improvement of lipid handling by macrophages in patients with cardiovascular disease. Simvastatin showed beneficial effects in the treatment of stroke‐prone hypertensive rats *via* decreasing macrophage infiltration and lipid deposition and inhibition of LOX‐1 expression 138. Similarly, pravastatin also reduced LOX‐1 expression in intimal macrophages and decreased lipid core size in the atherosclerotic plaques of Watanabe heritable hyperlipidaemic rabbits 139. In ApoE‐deficient mice, K‐604 and rimonabant, recently developed ACAT1 inhibitors, suppressed foam cell formation and reduced atherosclerosis 140, 141.

Pro‐protein convertase subtilisin/kexin type 9 (PCSK9) belongs to the family of PCSKs involved in the activation of peptide hormones and receptors from precursors. Specifically, PCSK9 decreases LDL cholesterol uptake by liver by stimulating degradation of LDL receptors 142. As a consequence, plasma LDL cholesterol (LDL‐C) concentrations become elevated which could increase cardiovascular risk. Increased expression of PCSKs was found in human and mouse atherosclerotic plaques suggesting the involvement of PCSKs in atherogenesis 143. To date, multiple PCSK9 inhibitors including low‐molecular inhibitors and monoclonal antibodies such as alirocumab (Aventis/Regeneron), bococizumab (Pfizer) and evolocumab (Amgen) have been developed 144. In animal models of atherosclerosis, PCSK9 inhibition resulted in significant lowering of plasma LDL‐C levels and diminished atherosclerosis progression 142. Clinical trials involving anti‐PCSK9 monoclonal antibodies showed a notable atheroprotective effect associated with cholesterol decrease, cardiac events and mortality 145.

In cultured human THP‐1 macrophages, PCSK9 expression was shown to be up‐regulated by oxLDL. Knock‐down of PCSK9 expression with PCSK9‐specific small interfering RNA resulted in the inhibition of oxLDL‐induced degradation of IκB‐α, an inhibitor of nuclear transcription factor NF‐κB that primes expression of a set of pro‐inflammatory genes 146. Indeed, PCSK9 inhibition prevents pro‐inflammatory activation of macrophages.

In addition to SRs, enzymes and transporters characterized in this review, it is necessary to investigate other biomolecules that could contribute to cholesterol handling in macrophages. Further studies are required to evaluate mechanisms that control expression and function of these biomolecules and investigate their involvement in cardiovascular disease. It is important to note here that in atherosclerosis, the disruption of lipid homoeostasis in macrophages, which leads to cholesterol accumulation and formation of foam cells, is only one of the well‐known mechanisms responsible for atherosclerotic plaque formation 1, 2, 3, 4, 5. Accumulated evidence indicate that the multifactorial inflammatory processes are responsible for the progression of atherosclerosis and that different cell types are simultaneously involved in all stages of plaque development 1, 2, 3, 4, 5.

## Disclosure

The authors declare no conflict of interest.
